# Adjuvant Radio-chemotherapy for extrahepatic biliary tract cancers

**DOI:** 10.1186/1471-2407-11-267

**Published:** 2011-06-24

**Authors:** Marta Bonet Beltrán, Arnaud D Roth, Gilles Mentha, Abdelkarim S Allal

**Affiliations:** 1 Servei d'Oncologia Radioteràpica, Consorci Sanitari de Terrassa, Institut Oncològic del Vallès (CST-HGC-CSPT), Ctra. Torrebonica s/n. 08227 Terrassa - Barcelona, Spain; 2 Radiation-Oncology, Geneva University Hospitals, Rue Gabrielle-Perret-Gentil 4, 1211 Genève 14, Switzerland; 3 Onco-Surgery and Surgery, Geneva University Hospitals, Rue Gabrielle-Perret-Gentil 4 1211 Genève 14, Switzerland; 4 Radiation-Oncology, HFR-Fribourg, Chemin des Pensionnats 2-6, 1752 Villars-sur-Glâne, Switzerland

## Abstract

**Background:**

Extrahepatic biliary duct cancers (EBDC) are uncommon malignancies characterized by a poor prognosis with high rate of loco-regional recurrence. The purpose of the present study is to assess the feasibility and the potential impact of adjuvant radiotherapy (RT) in a series of patients treated in one institution.

**Methods:**

Twenty three patients with non-metastatic bile duct cancer treated surgically with curative intent (4 gallbladder, 7 ampullary and 12 cholangiocarcinoma) received 3D conformal external beam RT to a median total dose of 50.4Gy. Concurrent chemotherapy based on 5-FU was delivered to 21 patients (91%). Surgical margins were negative in 11 patients (48%), narrow in 2 (9%), and microscopically involved in 8 (35%). Eleven patients (55%) had metastatic nodal involvement. The average follow-up time for all patients was 30 months (ranging from 3-98).

**Results:**

Acute gastrointestinal grade 2 toxicity (RTOG scale) was recorded in 2 patients (9%). Nausea or vomiting grade 1 and 2 was observed in 8 (35%) and 2 patients (9%) respectively. Only one patient developed a major late radiation-induced toxicity. The main pattern of recurrence was both loco-regional and distant (liver, peritoneum and/or lung). No difference was observed in loco-regional control according to the tumor location. The 5-year actuarial loco-regional control rate was 48.3% (67% and 30% for patients operated on with negative and positive/narrow/unknown margins respectively, p = 0.04). The 5-year actuarial overall survival was of 35.9% for the entire group (61.4% in case of negative margins and 16.7% in case of positive/narrow/unknown margins, p = 0.07).

**Conclusions:**

Postoperative RT with 50-60 Gy is feasible with acceptable acute and late toxicities. The potential benefit observed in our series may support the use of adjuvant RT in patients with locally advanced disease. Prospective randomized trials are warranted to confirm definitively the role of RT in this tumor location.

## Background

Biliary tract cancers are uncommon malignancies. Adenocarcinoma of the gallbladder occurs in 2 cases per 100.000 inhabitants [1], and cholangiocarcinoma and ampullary cancer in only 1.7 cases per 100.000 inhabitants. The main characteristic of these tumors is their poor prognosis [2].

To date, the only treatment providing a chance for cure is surgery. Despite an improvement in surgical techniques and the use of more aggressive surgical approaches, only a minority of patients (20-50%) are suitable for radical procedures [3]. In these resected patients, improved survival rates have been related to negative margin status [4]. Patients with initial stages and negative margins can reach a 5-year survival of 60-70% [5-7]. However, free margins are obtained in no more than one half of patients [8]. The main patterns of failure and some clinico-pathological prognostic factors have been identified in a few retrospective studies, suggesting that loco-regional recurrence is the most frequent site of relapse even after aggressive surgical procedures [9]. Due to this pattern, the addition of radiotherapy appears as a logical adjuvant treatment aiming to decrease loco-regional recurrence and improve survival.

To our knowledge, there is only one phase III randomized trial of adjuvant chemo-radiation involving patients with resected ampullary cancers [10]. This study evaluated a pool of periampullary tumors (ampulla, duodenum and distal bile duct cancers) and showed no benefit in adding adjuvant chemo-radiation to surgery. Thus, given the small number and the retrospective nature of published single institution studies, the impact of adjuvant RT has not been yet clarified.

In our institution, adjuvant RT is proposed to patients with resected extrahepatic biliary tract cancer presenting with unfavorable features. The aim of this study is to assess retrospectively the feasibility and the potential role of post-operative RT in a series of patients treated in a single institution.

## Methods

### Study design: cohort study

38 patients with non-metastatic biliary tract cancer were treated with radiotherapy in our institution (the Radiation Oncology Department, Geneva University Hospitals, HUG) between 1998 and 2004. The beginning of this period corresponds to the start of the routine use of 3D-CRT while the end was chosen to allow a longer follow-up after the treatment. We excluded twelve patients with unresectable disease and three with intrahepatic cholangiocarcinoma. The pretreatment patient and disease characteristics of the remaining 23 patients are shown in table 1. Stage classification was based on the pathological findings according to the 5th edition TNM/ American Joint Committee (AJCC) on Cancer staging system. There were 4 patients with gallbladder cancer, 7 with ampullaly cancer and 12 with cholangiocarcinoma. According to the recommendations of the HUG's Ethical Committee, written informed consent was obtained from all surviving patients.

**Table 1:** Tumor and patients characteristics

		Gallbladder [n = 4]	Extrahepatic cholangiocarcinoma [n = 12, 3 hilar, 9 distal]	Ampullary [n = 7]
Sex	male	1	9	4

	female	3	3	3

Age		66 [51-77]	62 [45-77]	63 [54-78]

AJCC stage 5^th ^edition	II	2	2	1

	III	2	0	6

	IVA	0	10	0

Margin status	negative	2	4	5

	narrow	1	0	1

	positive	0	7	1

	unknown	1	1	0

Nodal involvement	pN0	2	6	1

	pN1	2	3	6

	pN2	0	1	0

	unknown	0	2	0

5-FU based chemotherapy	yes	3	12	6

	no	1	0	1

Surgical procedures included laparoscopic cholecystectomy in 2 patients (9%), partial bile duct resection with or without hepatectomy in 4 (17%) and 3 (13%) patients respectively, and pancreatico-duodenectomy with or without partial gastrectomy in 6 (26%) and 7 (30%) patients respectively. Loco-regional lymph nodes were dissected with curative intent in 20 patients (87%). The mean removed nodes was of 11 (range 3-23). All tumors were adenocarcinoma with a high histological grade in the majority of cases. Stages III and IVA represented 78% of patients. Eleven patients (55%) had metastatic nodal involvement. The surgical margins were negative in 11 patients (47%), narrow in 2 patients (9%), microscopically involved in 8 (35%) and unknown in 2 (9%).

Target volumes were defined on the basis of clinical data, preoperative imaging and histopathological findings. All patients received 3-D conformal external beam RT (EBRT) to the tumor bed and regional lymphatic drainage according to the tumor location (*porta hepatis *and celiac nodes for the entire locations; the pericholedocal and duodenopancreatic nodes in gallbladder cancer; the peripancreatic in cholangiocarcinoma as well the para-aortic nodes in case of distal tumor location; and peripancreatic, superior mesenteric artery and para-aortic nodes in ampullary cancer). RT was delivered by standard fractionation of 1.8-2 Gy/day, 5 times a week to a median dose of 45 Gy (range 36-52 Gy), followed by a boost with reduced fields to a median total dose of 50.4 (rage 45-60 Gy). In only one patient with macroscopically positive margins (hepatic parenchyma), an intraluminal brachytherapy boost was performed (20 Gy in 4 fractions of 5 Gy with Ir-192 sources by percutaneous biliary catheterization) after 50.4 Gy of EBRT. Twenty patients received 5-FU based concomitant chemotherapy (5FU 500 mg/m2 the first and last 3 days of RT, 5FU-leucovorin or 5FU-cisplatinum) and one patient received a daily cisplatinum dose of 6 mg/m2. All patients had ECOG 1-2 performance status before treatment, and only two patients did not receive chemotherapy due to advanced age and comorbidities.

Statistical analysis was performed using the Statistical Package for the Social Sciences software (SPSS, Chicago, IL, USA). Survival was calculated according to the Kaplan-Meier method.

## Results

All patients completed the planned EBRT schedule without interruption. The mean time interval between surgery and the start of EBRT was 66.5 days. Acute and late radiation toxicities were assessed according to the Radiation Therapy Oncology Group (RTOG)/NCI-CTCAE v3.0 criteria [11]. Acute grade 2 abdominal pain and diarrhea was recorded in 2 patients (9%). Nausea or vomiting grade 1 and 2 was present in 8 (35%) and 2 (9%) patients respectively, mostly associated with concomitant 5-FU-based chemotherapy. No patient presented acute grade 3 or 4 toxicity. Only one patient developed a late grade 3 (RTOG) toxicity, consisting in an anastomotic stenosis which required a partial gastrectomy.

The median follow-up for all patients was 30 months (range 3-98). At last follow up 8 patients were alive (7 tumor-free), 14 deceased secondary to cancer failure and one was lost to follow-up 13 months after treatment. Loco-regional failure was defined as relapse in the site of the primary tumor and/or the regional lymph node areas, whereas peritoneal spread was considered as distant recurrence. We observed 4 in-field failures with local component, 4 patients with hepatic metastasis, and distant spread was observed in 8 (peritoneal, pulmonary and skin). Nine patients developed both loco-regional and distant spread, which therefore represents the main pattern of failure in our series.

**Figure 1:**
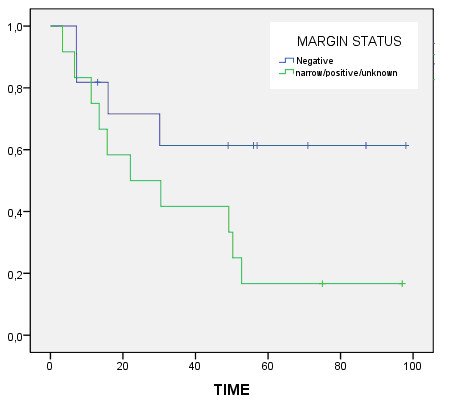
**Actuarial overall survival for patients according to the tumor resection margins**.

The 5-year actuarial loco-regional control rate was 48.3%. This figure was 67% and 30% for patients operated on with negative and positive/narrow/unknown margins respectively (p = 0.04). The 5-year actuarial overall survival was 35.9% for the entire group, 61.4% for patients with negative margins and 16.7% for patients with positive/narrow/unknown margins (p = 0.07) (figure 1). Patients with stage II (22%) had a median survival of 52 months compared to 22 months for patients with stage IV (p = 0.27). Patients with ampullary location had a non significant higher overall survival rate (57% vs 24.5%, p = 0.33).

## Discussion

Biliary tract cancers are rare diseases. The Geneva Cancer Registry has reported an incidence of 4.5 cases per 100,000 inhabitants, associated with a poor 5-year survival (12% in Switzerland) [2].

Complete surgical resection remains the only potential curative treatment. However, despite the improvement in the resectability rates in the last decades, less than 50% of patients would be treated with curative resections [12,13]. In a recent Japanese cohort study of patients with biliary tract cancers (ampullary, gallbladder and cholangiocarcinoma), the 5-year survival rates for patients with AJCC 2002 stages IA, IB, IIA, IIB, and III were 85%, 75%, 36%, 20%, and 0%, respectively [14].

In agreement with the WHO classification [15], cholangiocarcinoma, gallbladder cancer and ampullary cancer have been considered as extrahepatic biliary tract neoplasms. Some authors advocate classifying this third location separately since the 5-year survival of ampullary cancer can reach 80% in early stages [5][7][16]. Although in our series we have observed a better survival of patients with cancer of the ampulla of Vater, this difference was not statistically significant.

The benefit of adjuvant RT or chemoradiation has not yet been proved. Due to the rarity of this cancer, it has been difficult to design prospective randomized clinical trials. In the other hand, the limitation of the majority of single institution studies lies in their small sample sizes, their retrospective nature, and the fact that clinico-pathological features of the irradiated versus observed patients were frequently imbalanced. Concerning ampullary cancer, there is only one prospective trial namely the phase III EORTC trial published in 1999 [10]. In this study, no clear benefit was associated with the addition of chemoradiation to surgery for a set of tumors pertaining to the duodenum, the distal bile duct and the ampulla of Vater. However one should note that the study included a mixture of tumor location and the majority of patients had an early stage disease (60% of stages T1/T2 and/or node-negative). For gallbladder cancer, attempts to define the role of adjuvant RT have led to some analysis of the SEER (Surveillance, Epidemiology and End Results of the National Cancer Institute) population databases. Three studies have recently been published. The first, published by Mojica et al, [17] analyzed 3187 patients of whom 17% were treated with adjuvant radiotherapy. It was observed an increase in median survival with adjuvant treatment (14 versus 8 months, p < 0.001) for the subgroup of patients presenting with locally advanced disease and lymph node involvement. The second [18], analyzed a sample of 4180 patients (18% received adjuvant radiotherapy) where despite the lack of information about the margin status, surgical technique and/or the use of chemotherapy, the addition of adjuvant RT was associated with a benefit in overall survival, particularly for patients with positive lymph nodes. The third study [19] aimed to define independent prognostic factors influencing overall survival. The use of RT and lymph node involvement were identified as the main factors with hazard ratios of 0.78 (95% CI 0.70-0.87, p < 0.01) and 0.65 (95% CI 0.54-0.79, p < 0.01) respectively.

In most of the published single centre studies, patients with positive lymph nodes and/or positive margins represents more than 50% of the irradiated patients. The reported median survival ranges between 13 to 34 months [12][20-22]. We observed a median survival of 49 months for the entire cohort and a better 5-year survival for patients with negative margins (61.4% *vs *16.7% if positive, narrow and/or unknown).

Few studies have appraised patients' outcome according to loco-regional control, an essential goal when evaluating the indication of RT or assessing its efficacy. Reported rates of relapse range from 50% to 70% [12][23-27], and the recurrence rate has been correlated to the disease stage. Murakami et *al*. reported a recurrence rate of 9%, 20%, 60%, 83%, and 100% in stages IA, IB, IIA, IIB, and III respectively [14]. Generally loco-regional recurrence occurs in the liver hilum, bilio-enteric anastomosis, liver resection margins and retroperitoneal lymph nodes. However, the anatomic definition for loco-regional relapse is not well defined in the literature. It is reported by some authors as recurrences in the tumor bed and/or primary lymph nodes drainage [9], while others include the recurrences in the peritoneum, intra-hepatic or at the abdominal wall scar [26]. In agreement with the first definition, we have considered peritoneal spread as distant failure, and in our series the observed loco-regional relapse rate was about 50%, which is consistent with the reported rates in the literature.

In our institution, the policy during the trial period was to administer concurrent 5-FU based chemotherapy. However, the role of chemotherapy alone or in combination with radiotherapy remains controversial. 5-FU has been frequently used in a combined modality approach because of its potential radiosensitivity [24][28,29]. Other chemotherapeutic agents such as gemcitabine (alone or combined with fluoropirimidins or oxaliplatin) demonstrated a good palliation effect in advanced tumor stages [30,31]. In the adjuvant setting, a benefit for patients with gallbladder cancer was shown in one Japanese randomized trial [32].

Acute toxicity related to adjuvant radiotherapy (with or without chemotherapy) is usually slight to moderate, and concerns mainly gastrointestinal symptoms such as nausea, vomiting or diarrhea. Doses up to 50-54 Gy at standard fractionation are normally well tolerated and toxicity rarely exceeds RTOG grades 1-2. Late bleeding secondary to gastric or duodenal ulcer and necrosis of the anastomosis requiring surgery are uncommon, with a reported incidence in the literature of less than 4% [22][33]. In the present series, only one patient required a partial gastrectomy for a late toxicity potentially radiation related. Gerhards et al [23] reported the toxicity of three post-operative approaches: observation, EBRT and EBRT plus brachytherapy. The toxicity profile was acceptable with the addition of EBRT, while the addition of a brachytherapy boost increased significantly local toxicity. In our study, the patient who developed an anastomotic stricture was treated only with EBRT.

## Conclusions

Our study reports the first retrospective data on Swiss population treated with postoperative RT after radical surgery in patients with EHBDC. Radiation doses of 50-60 Gy at standard fractionation in combination with 5-FU based chemotherapy can be administered with acceptable acute and late toxicities.

While we observed that patients with positive surgical margins had a lower survival and loco-regional control, the low incidence of severe toxicity and the potential benefit observed in our series may support the indication for adjuvant RT in patients with locally advanced disease.

However, given the poor prognosis of this cancer despite the use of adjuvant therapy, an improvement of the radiation techniques with dose escalation and/or the combination with new chemotherapeutic agents should be considered in the frame of prospective trials.

## List of abbreviations

In alphabetic order:

AJCC: American Joint Committee on Cancer; EBRT: External Beam Radiotherapy; EHBDC: Extrahepatic Bile Duct Cancer; EORTC: European Organization for Research and Treatment of Cancer; HR: Hazard Ration; RT: Radiotherapy; SEER: Surveillance, Epidemiology and End Results; WHO: World health organization.

## Competing interests

The authors don't have any financial interests or connections, direct or indirect, or other situations that might raise the question of bias in the work reported or the conclusions, implications, or opinions stated including pertinent commercial or other sources of funding for the individual authors or for the associated departments, personal relationships, or direct academic competition.

## Authors' contributions

All the authors have made substantial contributions to conception and design, or acquisition of data, or analysis and interpretation of data; have been involved in drafting the manuscript or revising it critically for important intellectual content; and have given final approval of the version to be published.

In addition, MBB has appropriately coordinated the study, managed the database, and focused on radiotherapy treatment, toxicities potentially related and follow-up of the patients; ADR has focused on chemotherapy treatment, toxicities potentially related and follow-up of the patients, as staff in charge in the period; GM has focused on surgical treatment, toxicities potentially related and follow-up of the patients, as staff in charge in the period; and ASA has focused on radiotherapy treatment, toxicities potentially related and follow-up of the patients, as staff in charge in the period.

## Pre-publication history

The pre-publication history for this paper can be accessed here:

http://www.biomedcentral.com/1471-2407/11/267/prepub
